# An untargeted metabolomic analysis of acute AFB1 treatment in liver, breast, and lung cells

**DOI:** 10.1371/journal.pone.0313159

**Published:** 2025-01-30

**Authors:** Heidi H. Cao, Sabrina Molina, Susan Sumner, Blake R. Rushing

**Affiliations:** 1 Department of Nutrition, University of North Carolina at Chapel Hill, Chapel Hill, NC, United States of America; 2 Nutrition Research Institute, University of North Carolina Chapel Hill, Kannapolis, NC, United States of America; Murdoch University, AUSTRALIA

## Abstract

Aflatoxin B1 (AFB1) is a class 1 carcinogen and mycotoxin known to contribute to the development of hepatocellular carcinoma (HCC), growth impairment, altered immune system modulation, and malnutrition. AFB1 is synthesized by Aspergillus flavus and is known to widely contaminate foodstuffs, particularly maize, wheat, and groundnuts. The mechanism in which AFB1 causes genetic mutations has been well studied, however its metabolomic effects remained largely unknown. A better understanding of how AFB1 disrupts metabolism would provide insight into how this mycotoxin leads to carcinogenesis, growth impairment, and/or immunomodulation, and may reveal potential targets for pharmacological or nutritional interventions to protect against these effects. The current study evaluated the metabolomic effects of various doses (2.5 μM, 5 μM, 10uM) of AFB1 treatment to HepG2 (liver), MDA-MB-231 (breast), and A549 (lung) cells. Treated and control cells’ metabolomic profiles were evaluated via ultra-high performance liquid chromatography-high resolution mass spectrometry (UHPLC-HRMS). Univariate and multivariate analyses revealed significant alterations in metabolite concentrations from each dose of AFB1 treatment in each cell type. Pathway analysis was then used to understand broader biochemical functions affected by AFB1 treatment in each cell type. HepG2 cell pathway analyses revealed significant pathway perturbations in lipid metabolism, carnitine synthesis, catecholamine biosynthesis, purine metabolism, and spermidine and spermine biosynthesis. Analysis of A549 cells found a greater emphasis of perturbations on various amino acids along with lipid synthesis-related pathways, and catecholamine biosynthesis. Finally, analysis of treated MDA-MB-231 cells found spermidine and spermine biosynthesis, carnitine synthesis, plasma membrane-related pathways (phosphatidylcholine synthesis and alpha linolenic acid and linoleic acid metabolism), and various amino acid metabolism pathways to be most affected. These highlighted pathways should be targeted in future investigations to evaluate their potential in mitigating or preventing the development of negative health effects associated with AFB1 exposure.

## Introduction

Aflatoxin B1 (AFB1) is the most toxic member of the aflatoxins, a class of mycotoxins produced by the fungus *Aspergillus flavus* [1]. AFB1 has been widely established as a causative agent in hepatocellular carcinoma (HCC) and is officially deemed a group 1 human carcinogen by the International Agency for Research on Cancer [1]. AFB1 has been found to contribute to 4.6–28.2% of HCC cases globally, partly due to a synergistic interaction with hepatitis B virus infection [2–5]. In addition, AFB1 has been shown to play a role in other detrimental health effects such as growth suppression, immune system modulation, and malnutrition which may be linked to liver or intestinal damage induced by AFB1 [6–9]. *A*. *flavus* contaminates essential food supplies (rice, corn, groundnuts, etc.) with AFB1, especially under high humidity and heat [10]. Due to their inability to be destroyed by standard cooking or industrial processing practices, strict monitoring of AFB1 in food sources is essential to prevent exposure to this toxin [9,11]. AFB1 levels are restricted in variable amounts depending on the country. The maximum allowed level (MAL) ranges from 2 ug/kg in Romania to 20 ug/kg in the United States, and countries in regions with high humidity and heat or inadequate climate-controlled storage are especially vulnerable to AFB1 contamination [12–14]. Thus, contamination and subsequent exposure to AFB1 is inevitable for certain populations. Therefore, an understanding of the mechanisms in which AFB1 causes its negative health effects is imperative to uncovering interventions to protect these populations.

The main mechanism of action of AFB1 that contributes to its carcinogenicity is triggered when it is metabolized by cytochrome P450 enzymes, particularly CYP3A4 and CYP1A2, to form aflatoxin B1-8,9-epoxide (AFBO), a highly reactive metabolite that forms AFB1-N7-guanine adducts when reacting with cellular DNA. AFB1-N7-guanine can spontaneously be converted to a decyclized AFB1-formamidopyridine adduct (AFB1-FAPy) or the guanine residue undergoes depurination and leaves an apurinic (AP) site. Both outcomes result in forms of DNA mutations [1,15,16]. A common form of mutation due to AFB1 exposure found to be implicated in HCC formation is a G→T transversion causing the mutation R249S in the p53 protein, a widely known tumor suppressor protein [17]. AFBO can also spontaneously form AFB1 dihydrodiol which readily interconverts with its open ring dialdehyde form, AFB1 dialdehyde. The latter form can react with primary amine groups on proteins and is presumed to contribute to a pro-inflammatory and pro-proliferative state in tissues associated with carcinogenesis [9,18]. Furthermore, AFBO can directly bind to RNA and protein and disrupt their normal functions [9,15]. Other metabolites or derivatives of AFB1 can form spontaneous adducts with cellular structures, notably aflatoxin M1 (AFM1) and aflatoxin B2a (AFB2a). AFM1 was found to have a similar mechanism as AFB1 in forming adducts with DNA after epoxidation following biotransformation by cytochrome P450s. However, AFM1 forms epoxides much less readily as compared to AFB1 and has been shown to be less mutagenic [9,19,20]. AFB2a binds to primary amines on amino acids, which has been shown to inhibit the activity of proteins such as deoxyribonucleases. AFB2a is also unique in its ability to form an adduct with phospholipids by binding to phosphoethanolamine head groups [21].

In addition to HCC, AFB1 exposure has been illustrated to participate in the development of other detrimental health disorders such as malnutrition, growth impairment, and immune modulation [6–9]. Multiple studies have found higher levels of aflatoxin biomarkers in kwashiorkor, marasmus, and marasmic kwashiorkor patients [7,22–24]. It has been hypothesized that AFB1 interferes with metal bioavailability, lipogenesis, renal function, and parathyroid metabolism [25–28]. Downregulation of the vitamin D receptor by AFB1 has been supported by *in vitro* studies [29]. Enteropathy, including impaired intestinal function and absorption, has been observed to correlate with childhood malnutrition and AFB1 exposure [30–32]. Taken together, this suggests a mechanism in which AFB1 causes malnutrition as well as growth impairment, possibly by affecting metabolic functions. Finally, impairment of immune function has been seen following AFB1 exposure by a reduction in immunoglobulins, anti-inflammatory cytokines, lymphocyte activation, and general suppressed humoral immunity in animal models [6,33–35]. Some studies do indicate that this effect may only be true for lower doses of AFB1 and higher doses cause greater activation of immune functions [36].

Altogether, these studies indicate that the toxic effects of AFB1 exposure are widespread and not all effects have a clearly defined mechanism. Moreover, the effects of AFB1 are likely to be different by cell type due to differences in the expression of enzymes that metabolize AFB1. While the mechanism of all AFB1-related toxicities is not clear, evidence suggests that metabolic disruptions may play a role, particularly in growth impairment and malnutrition. A comprehensive investigation into the metabolic effects of AFB1 exposure could provide valuable insight into understanding potential mechanisms that underlie AFB1 toxicity, and may point towards intervention strategies to prevent or mitigate those effects. Metabolomics enables the elucidation of changes in metabolism that occur in response to exposures which can be used to elucidate mechanisms and potential interventions to mitigate their negative effects [37]. Identification of affected pathways and metabolites could better inform the understanding of how AFB1 adversely affects the body, how it contributes to various health issues, and how targeted pharmacological or nutritional therapies can be developed to combat the effects of AFB1 exposure. We present herein an untargeted metabolomic analysis of acute AFB1 treatment in HepG2, MDA-MB-231, and A549 cell lines in order to investigate metabolic pathways that are affected by AFB1 in liver, breast, and lung cells.

## Materials and methods

### Chemical reagents

Optima grade solvents (water with 0.1% formic acid and methanol with 0.1% formic acid) and fetal bovine serum (FBS) were purchased from Fisher Scientific (Waltham, MA, USA). Phosphate-buffered saline (PBS) and Dubelcco’s Modified Eagle Medium (DMEM) with high glucose were purchased from Gibco (Grand Island, NY, USA). AFB1 was purchased from Cayman Chemical (Ann Arbor, MI, USA). A549, HepG2, and MDA-MB-231 cell lines were purchased from the American Type Culture Collection (ATCC) (Manassas, VA, USA).

### Cell culture

A549, HepG2, and MDA-MB-231 cells were cultured in DMEM supplemented with 10% FBS, 2 mM glutamine, 50 U/mL penicillin, and 50 μg/mL streptomycin. Cells were plated in 6 well plates and grown to 80% confluency and were treated with AFB1 at doses of 0, 2.5, 5, and 10 μM. Dimethyl sulfoxide (DMSO) was used as the vehicle with a concentration of 0.1% for all treatments. All treatments were performed for 24 h (n = 4 per treatment). The range of treatment concentrations were based off of our previous work which showed bioactivity of AFB1 in liver cells at similar doses [38].

### Metabolite extraction

After treatment, metabolites were extracted from cell samples as described previously [39–43]. Briefly, treatment media was aspirated, and cells were washed with 2 mL of ice-cold PBS followed by the addition of 1 mL of an ice-cold solution of 80% methanol and 20% water. Cells were detached using cell scrapers and vortexed at 5000 rpm for 10 min. Protein concentration was measured by a bicinchoninic acid (BCA) assay, and additional 80% methanol was added to each tube to normalize for protein concentration. Samples were centrifuged at 16,000× g at 4°C for 10 min and supernatants were transferred to autosampler vials for analysis by ultra-high-pressure liquid chromatography–high-resolution mass spectrometry (UHPLC-HRMS). Quality control study pools (QCSP) were created by combining 50 μL of each sample into a single mixture. Three separate QCSPs were made for each cell line. Method blanks were created by adding 500 μL of 80% methanol to empty tubes and were processed in an identical manner as the study samples. All samples for a given cell line were derived from the same cryovial.

### UHPLC-HRMS metabolomics data acquisition, preprocessing, and multivariate analysis

The metabolomics data in this study were generated using established methods as previously described [39–45]. In brief, the data were collected using a Vanquish UHPLC system coupled to a Q Exactive™ HF-X Hybrid Quadrupole-Orbitrap Mass Spectrometer (Thermo Fisher Scientific, San Jose, CA, USA). Chromatographic separation was achieved with an HSS T3 C18 column (2.1 × 100 mm, 1.7 μm, Waters Corporation) held at a constant temperature of 50°C. The mobile phases consisted of water with 0.1% formic acid (A) and methanol with 0.1% formic acid (B). The mobile phase gradient was initiated with 2% B, increased to 100% B over 16 minutes, and then maintained at 100% for 4 minutes, all at a flow rate of 400 μL/min. Mass spectral data were acquired using a data-dependent acquisition mode in positive polarity within a mass range of 70–1050 m/z. Data-dependent acquisition mode was used to fragment the top 20 ions in each spectra. To monitor instrument stability and data quality, quality control samples (QCSP) and blank injections were interspersed throughout the sample set during data acquisition, and each sample was analyzed with a 5 μL injection volume. The acquired UHPLC-HRMS raw data were subsequently processed using Progenesis QI (version 2.1, Waters Corporation, MA, USA) for alignment, peak picking, and deconvolution. Background noise was eliminated by filtering out peaks with higher average abundances in the blank injections compared to the QCSP injections. Data normalization was performed utilizing a reference sample from the QCSP, employing the "normalize to all" function within the Progenesis software.

### Compound identification/annotation

Peaks were matched to an in-house library of reference standards or public mass spectral databases from the National Institute of Standards and Technology (NIST), the Human Metabolome Database (HMDB), and METLIN. The matching criteria encompassed multiple parameters, including retention time (RT, within ±0.5 minutes for in-house library matches), exact mass (MS, within <5 ppm), and fragmentation pattern (MS/MS, with a similarity score > 30). An ontology system was used to denote the evidence basis for each metabolite assignment, with the following designations: OL1 indicated a match to the in-house library for retention time, exact mass, and MS/MS; OL2a indicated an in-house match to the in-house library for retention time and exact mass; OL2b referred to a match to the in-house library for exact mass and MS/MS; PDa indicated a match to public databases for exact mass and MS/MS; PDb represented a public database match for exact mass and theoretical MS/MS (e.g., HMDB); PDc was used when there was a match to public databases for exact mass and isotopic similarity, and PDd signified a match to public databases for exact mass only.

### Multivariate and Univariate Statistical Analysis

Following normalization and filtration, data were imported into SIMCA 16 (Sartorius Stedim Data Analytics AB, Umeå, Sweden). Pareto scaling was applied to all peaks for multivariate analysis. Principal component analysis (PCA) plots were built to evaluate data quality and unsupervised sample grouping, while orthogonal partial least squares-discriminant analysis (OPLS-DA) plots were employed as a supervised method to assess the differentiation of metabolomes between vehicle-treated cells and the experimental groups. Variable importance to projection (VIP) scores were calculated for each detected peak using the OPLS-DA models. All models were built using a 7-fold cross validation to assess the predictive ability of the model (Q2). Fold changes and p-values (using Students’ t-test) were calculated for each peak. P-values were not adjusted for multiple testing due to the exploratory, rather than confirmatory, nature of this study [46].

### Pathway analysis

In-house matched peaks were uploaded to MetaboAnalyst 5.0 [47] for pathway analysis. Correlations were performed using the “Statistical Analysis [metadata table]” module. Concentration of AFB1 treatment (0 uM, 2.5 μM, 5 μM, 10 μM) was categorized as a continuous variable under the covariate study design. A correlation and partial correlation analysis was performed using the correlation measure Pearson r. Metabolites with a p-value < 0.05 for each cell type were used for Pathway Analysis. Fold changes and two-sided Student’s t-test were calculated between each individual treatment dosage group with its control in all cell types using the volcano plot function in the “Statistical Analysis [one factor]” module in MetaboAnalyst. Metabolites with a p-value < 0.1 were used for Pathway Analysis. Significant compounds from each analysis were imported into MetaboAnalyst 5.0’s “Pathway Analysis” module to identify significantly perturbed metabolic pathways. The Small Molecule Pathway Database (SMPDB) pathway library was used as the pathway database and pathways with a p<0.1 were considered significant.

## Results

**Fig 1:**
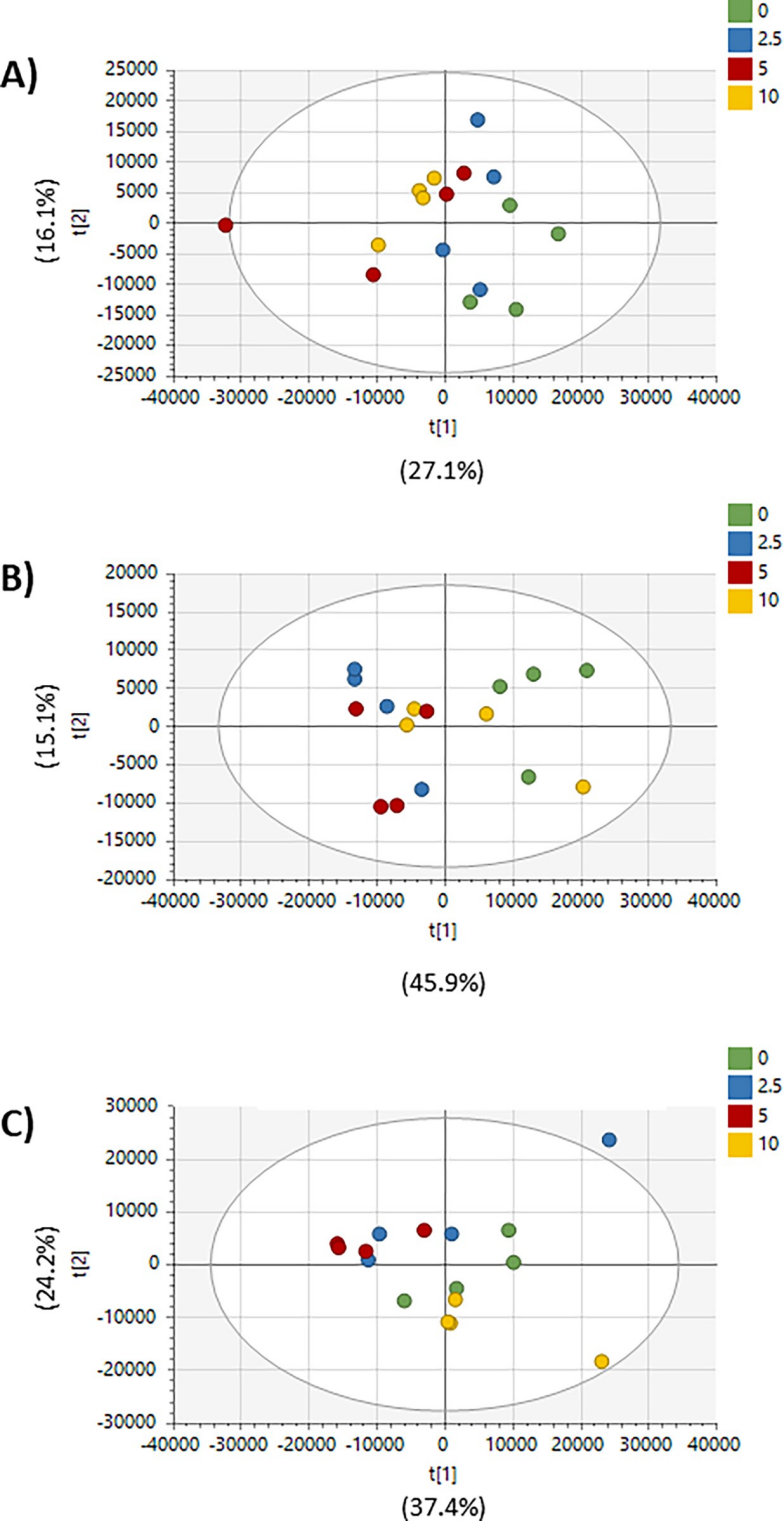
Unsupervised multivariate analysis of lung, liver, and breast metabolomes following AFB1 treatment. Principal component analysis (PCA) of A) A549, B) HepG2, and C) MDA-MB-231 cells using all metabolomics features.

**Fig 2:**
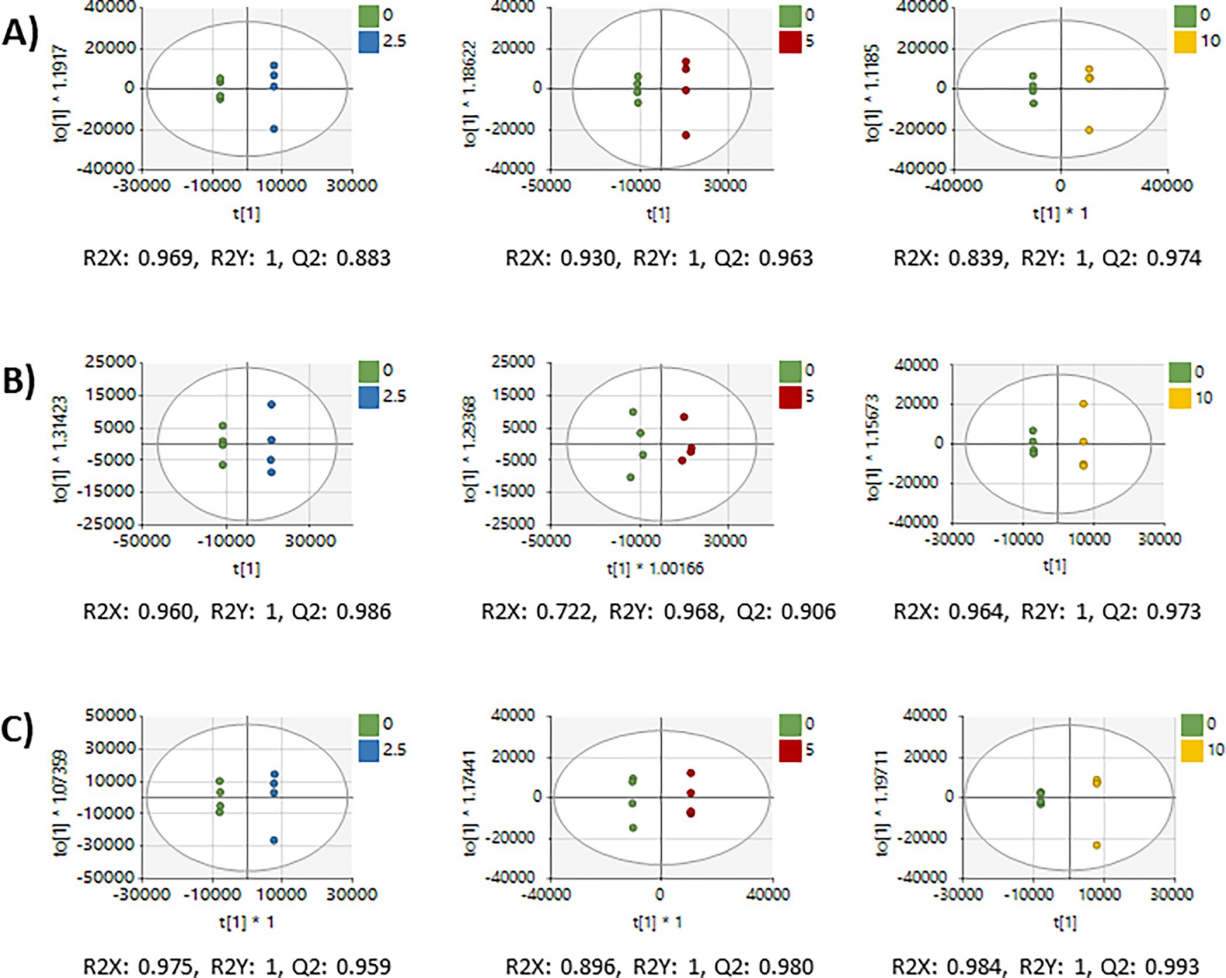
Supervised multivariate analysis of lung, liver, and breast metabolomes comparing vehicle and each AFB1 dose. OPLS-DA of 0 vs 2.5 μM (left), 0 vs 5 μM (middle), and 0 vs 10 μM (right) of A) A549, B) HepG2, and C) MDA-MB-231 cells using all metabolomics features.

After data preprocessing, 20,315 features remained in the untargeted dataset. PCA of all peaks showed tight clustering of each cell line’s QCSPs in the center of the study samples, a common benchmark of metabolomics data quality (S1 Fig) [48]. Additionally, PCA showed a moderate separation of dosage levels in each cell line (Fig 1A–1C). Supervised multivariate analysis using OPLS-DA showed strong and reproducible separation of each dose level from the vehicle control in each cell line (R2X, Q2 > 0.7) (Fig 2A–2C). OPLS-DA models were used to calculate VIP values for each peak. Fold changes and p-values were also calculated for each peak using normalized peak intensities for each dose-vehicle comparison for each cell line (S1 Table). Matching peaks to compounds revealed 566 peaks matched to the in-house library and 14,898 peaks matched to public databases.

**Fig 3:**
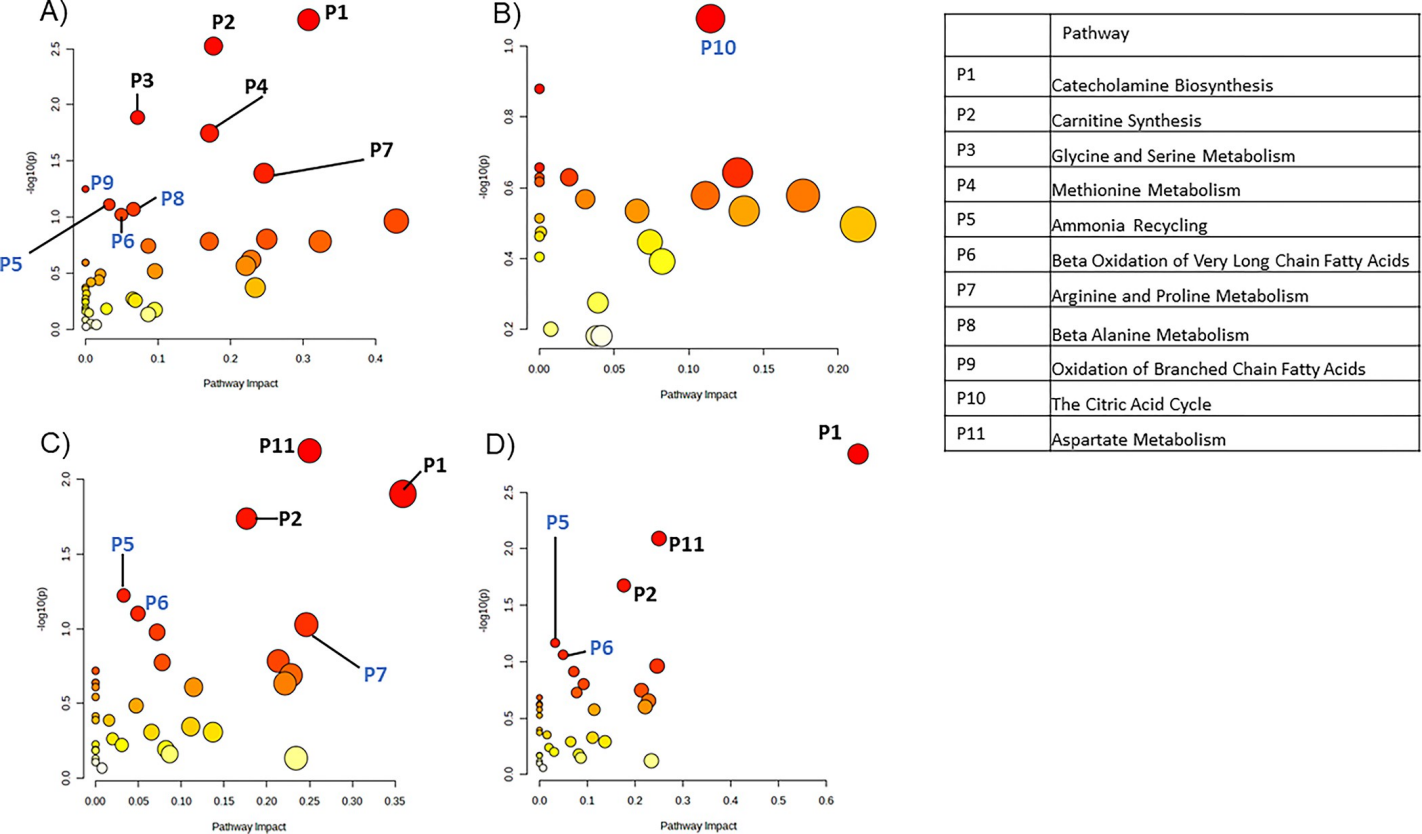
Pathway analysis reveals metabolic processes affected by AFB1 treatment in A549 cells. A) Pathway analysis of metabolites significantly correlated against AFB1 treatment levels in A549 cells using MetaboAnalyst’s metadata analysis module. B) Pathway analysis of significantly changed metabolites found in A549 cells treated with 2.5 μM, C) 5 μM, and D) 10 μM compared to the vehicle control. Blue pathway labels indicate pathways with p-values between 0.05 and 0.1. Black pathway labels indicate p<0.05.

Correlation analysis between peak intensities and AFB1 dose levels was then performed using only in-house matched metabolites. For A549 samples, this correlation analysis yielded 99 in-house matched metabolites that significantly correlated with AFB1 dose levels (p<0.05). These significant metabolites were subsequently imported into MetaboAnalyst’s pathway analysis, identifying nine significant pathways (p<0.1) which included catecholamine biosynthesis, carnitine biosynthesis, glycine and serine metabolism, methionine metabolism, arginine and proline metabolism, oxidation of branched chain fatty acids, ammonia recycling, beta-alanine metabolism, and beta oxidation of very long chain fatty acids (Fig 3A and Table 1). Pairwise pathway comparisons between the vehicle and each dose level were also performed using metabolites with p<0.1. Comparison of the vehicle with the 2.5 μM group produced 82 significant in-house matched metabolites which produced one significant pathway (Fig 3B and Table 1). Comparison of the vehicle and 5 μM groups showed 147 significant in-house matched metabolites which led to six significant pathways (Fig 3C and Table 1). The 10 μM treatment versus vehicle comparison found 160 significant in-house matched metabolites, which produced five significant pathways (Fig 3D and Table 1). Catecholamine biosynthesis, carnitine synthesis, beta oxidation of very long chain fatty acids, arginine and proline metabolism, and ammonia recycling were found to be significant in higher dosage treatment group analyses (5 μM and 10 μM) as well as in the correlation analysis. The only significant pathway yielded by the 2.5 μM treated cells was the tricarboxylic acid cycle.

**Table 1:** Significant pathways (p<0.1) and their corresponding p-values identified in AFB1-treated A549 cells.

Analysis Group	Pathway	p-Value
Correlation Analysis	Catecholamine Biosynthesis	0.0017522
	Carnitine Synthesis	0.003002
	Glycine and Serine Metabolism	0.012952
	Methionine Metabolism	0.017863
	Arginine and Proline Metabolism	0.040517
	Oxidation of Branched Chain Fatty Acids	0.056059
	Ammonia Recycling	0.076988
	Beta-Alanine Metabolism	0.084639
	Beta Oxidation of Very Long Chain Fatty Acids	0.09469
Vehicle vs 2.5 μM	Citric Acid Cycle	0.083481
Vehicle vs 5 μM	Aspartate Metabolism	0.0064384
	Catecholamine Biosynthesis	0.01251
	Carnitine Synthesis	0.018294
	Ammonia Recycling	0.05989
	Beta Oxidation of Very Long Chain Fatty Acids	0.079168
	Arginine and Proline Metabolism	0.093861
Vehicle vs 10 μM	Catecholamine Biosynthesis	0.0014465
	Aspartate Metabolism	0.008099
	Carnitine Synthesis	0.021101
	Ammonia Recycling	0.068185
	Beta Oxidation of Very Long Chain Fatty Acids	0.086816

**Fig 4:**
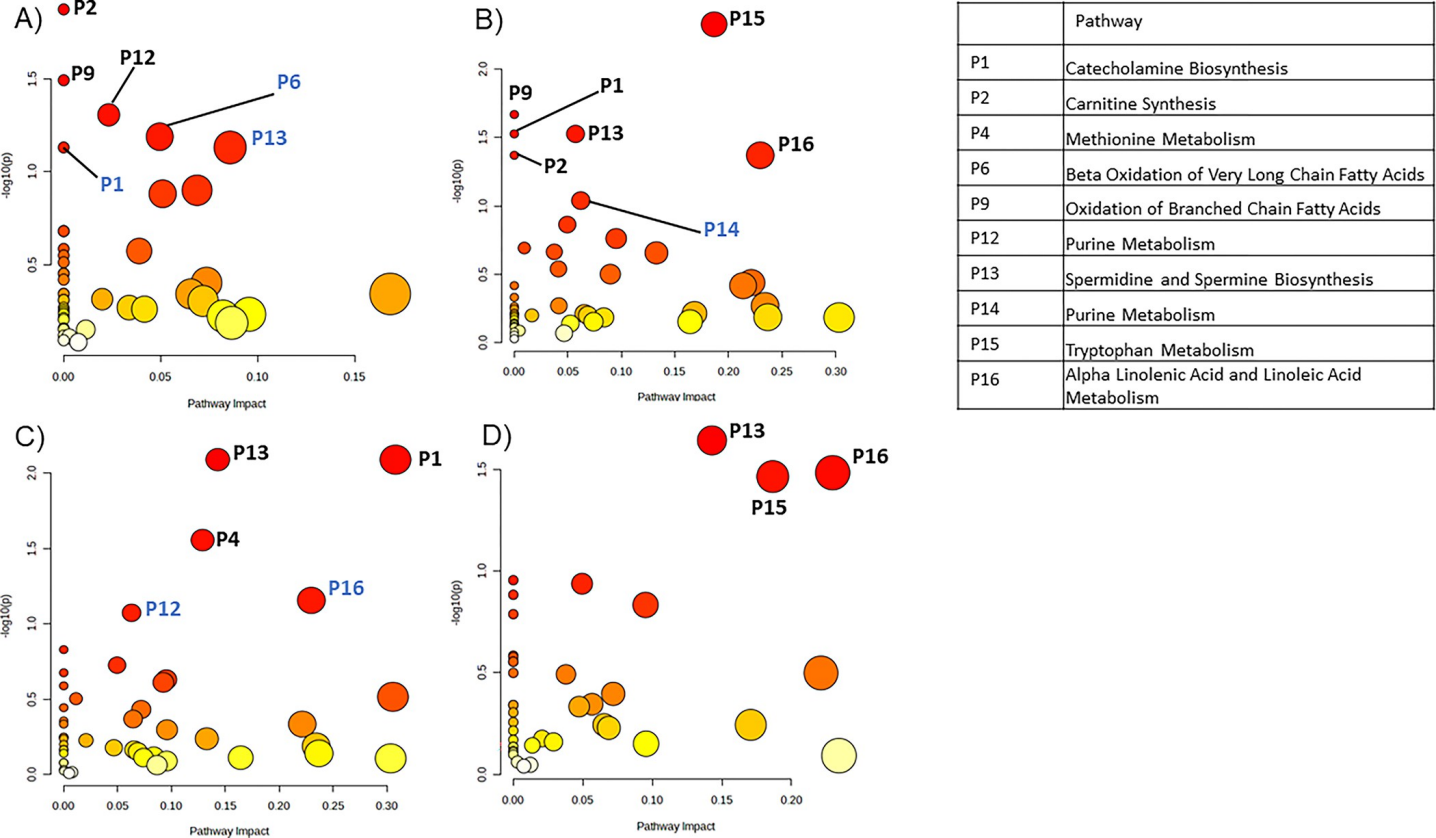
Pathway analysis reveals metabolic processes affected by AFB1 treatment in HepG2 cells. A) Pathway analysis of metabolites significantly correlated against AFB1 treatment levels in HepG2 cells using MetaboAnalyst’s metadata analysis module. B) Pathway analysis of significantly changed metabolites found in HepG2 cells treated with 2.5 μM, C) 5 μM, and D) 10uM compared to the control. Blue pathway labels indicate pathways with p-values between 0.05 and 0.1. Black pathway labels indicate p<0.05.

For HepG2 cells, correlation of metabolite intensities with AFB1 dose levels revealed 93 significant metabolites (p<0.05) which revealed six significant pathways (p<0.1) in pathway analysis (Fig 4A and Table 2). Pairwise comparisons of the 2.5 μM treatment with vehicle-treated HepG2 cells revealed 193 significant matched metabolites which corresponded to seven significant pathways (Fig 4B and Table 2). The analysis of the 5 μM treatment group found 218 significant matched metabolites which revealed five significant pathways (Fig 4C and Table 2). The 10 μM treatment group analysis generated 189 significant matched metabolites and three significant pathways (Fig 4D and Table 2). Major pathways found across multiple analyses of treated HepG2 cells include spermidine and spermine biosynthesis, alpha linolenic acid and linoleic acid metabolism, purine metabolism, and catecholamine biosynthesis.

**Table 2:** Significant pathways (p<0.1) and their corresponding p-values identified in AFB1-treated HepG2 cells.

Analysis Group	Pathway	p-Value
Correlation Analysis	Carnitine Synthesis	0.013358
	Oxidation of Branched Chain Fatty Acids	0.032128
	Purine Metabolism	0.049297
	Beta Oxidation of Very Long Chain Fatty Acids	0.064612
	Catecholamine Biosynthesis	0.073867
	Spermidine and Spermine Biosynthesis	0.073867
Vehicle vs 2.5 μM	Tryptophan Metabolism	0.004733
	Oxidation of Branched Chain Fatty Acids	0.021551
	Catecholamine Biosynthesis	0.02985
	Spermidine and Spermine Biosynthesis	0.02985
	Carnitine Synthesis	0.042762
	Alpha Linolenic Acid and Linoleic Acid Metabolism	0.042762
	Purine Metabolism	0.091532
Vehicle vs 5 μM	Spermidine and Spermine Biosynthesis	0.0081301
	Catecholamine Biosynthesis	0.0081301
	Methionine Metabolism	0.027779
	Alpha Linolenic Acid and Linoleic Acid Metabolism	0.069745
	Purine Metabolism	0.084339
Vehicle vs 10 μM	Spermidine and Spermine Biosynthesis	0.022681
	Alpha Linolenic Acid and Linoleic Acid Metabolism	0.032731
	Tryptophan Metabolism	0.034196

**Fig 5:**
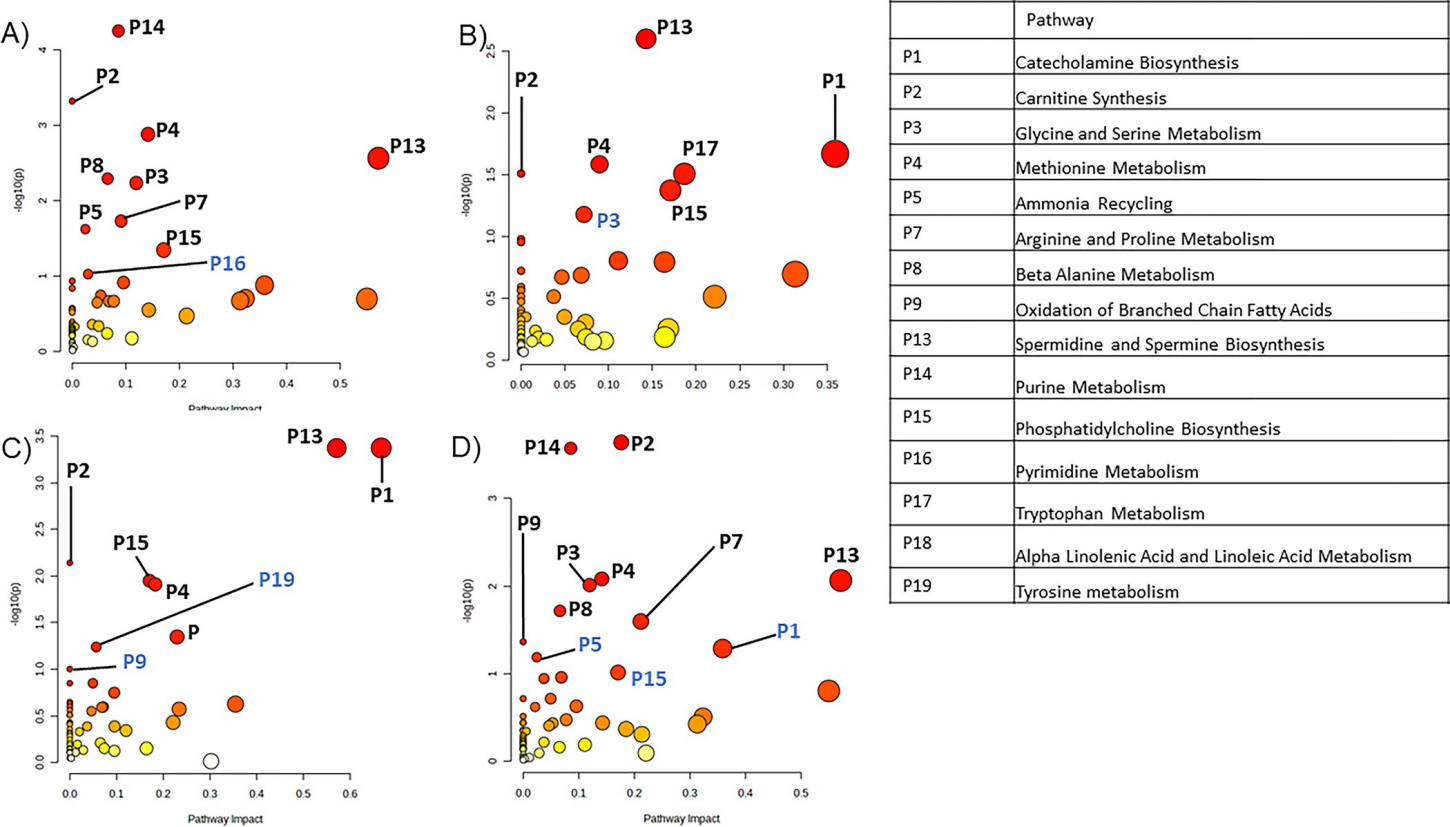
Pathway analysis reveals metabolic processes affected by AFB1 treatment in MDA-MB-231 cells. A) Pathway analysis of metabolites significantly correlated against AFB1 treatment levels in MDA-MB-231 cells using MetaboAnalyst’s metadata analysis module. B) Pathway analysis of significantly changed metabolites found in MDA-MB-231 cells treated with 2.5 μM, C) 5 μM, and D) 10 μM compared to the control. Blue pathway labels indicate pathways with p-values between 0.05 and 0.1. Black pathway labels indicate p<0.05.

Lastly, for MDA-MB-231 cells, correlation analyses of the concentrations of matched features and AFB1 treatment level found 146 significant matched metabolites (p<0.05) which corresponded to ten pathways with p<0.1 (Fig 5A and Table 3). When comparing metabolite intensities between vehicle and 2.5 μM AFB1 treated MDA-MB-231 cells, 141 matched metabolites with p<0.1 were identified. Inputting these metabolites into pathway analysis, seven significant pathways (p<0.1) were revealed (Fig 5B and Table 3). The same statistical and pathway analysis with MDA-MB-231 cells treated with 5 μM AFB1 generated 194 significant in-house matched metabolites which yielded eight significant pathways (Fig 5C and Table 3). Lastly, the 10 μM AFB1 treatment resulted in 208 significant matched metabolites and 11 pathways (Fig 5D and Table 3). Some notable pathways that were affected aligned with those found in A549 and HepG2, such as spermidine and spermine biosynthesis, carnitine synthesis, and catecholamine biosynthesis. Oxidation of branched chain fatty acids were seen to be significant in some analyses of every cell type examined. Methionine metabolism, phosphatidylcholine biosynthesis, glycine and serine metabolism were consistently and uniquely found to be significant in MDA-MB-231 cells following AFB1 treatment.

**Table 3:** Significant pathways (p<0.1) and their corresponding p-values identified in AFB1-treated MDA-MB231 cells.

Analysis Group	Pathway	p-Value
Correlation Analysis	Purine Metabolism	5.57E-05
	Carnitine Synthesis	0.00047428
	Methionine Metabolism	0.0013066
	Spermidine and Spermine Biosynthesis	0.0027109
	Beta-Alanine Metabolism	0.0050588
	Glycine and Serine Metabolism	0.0057772
	Arginine and Proline Metabolism	0.018456
	Ammonia Recycling	0.023667
	Phosphatidylcholine Biosynthesis	0.044748
	Pyrimidine Metabolism	0.093394
Vehicle vs 2.5 μM	Spermidine and Spermine Biosynthesis	0.0024954
	Catecholamine Biosynthesis	0.021379
	Methionine Metabolism	0.025987
	Carnitine Synthesis	0.030897
	Tryptophan Metabolism	0.031021
	Phosphatidylcholine Biosynthesis	0.042302
	Glycine and Serine Metabolism	0.066297
Vehicle vs 5 μM	Spermidine and Spermine Biosynthesis	0.00042168
	Catecholamine Biosynthesis	0.00042168
	Carnitine Synthesis	0.0072082
	Phosphatidylcholine Biosynthesis	0.011221
	Methionine Metabolism	0.012269
	Alpha Linolenic Acid and Linoleic Acid Metabolism	0.044939
	Tyrosine Metabolism	0.057689
	Oxidation of Branched Chain Fatty Acids	0.099538
Vehicle vs 10 μM	Carnitine Synthesis	0.00022882
	Purine Metabolism	0.00026723
	Methionine Metabolism	0.0082763
	Spermidine and Spermine Biosynthesis	0.0086103
	Glycine and Serine Metabolism	0.0097327
	Beta-Alanine Metabolism	0.019161
	Arginine and Proline Metabolism	0.025288
	Oxidation of Branched Chain Fatty Acids	0.043149
	Catecholamine Biosynthesis	0.051514
	Ammonia Recycling	0.064865
	Phosphatidylcholine Biosynthesis	0.096749

## Discussion

AFB1 has been identified as an international hazard to human health due to its demonstrated role in HCC as well as its potential role in malnutrition, growth impairment, and immune modulation. Yet, its precise mechanism of action in some of these effects is still not completely understood. Using untargeted metabolomics, we sought to further investigate the effect of acute AFB1 treatment on cellular metabolism and how this differs between lung (A549), liver (HepG2), and breast (MDA-MB-231) cells. A549 and HepG2 cells were selected for our investigation due to AFB1’s established carcinogenic effect on liver and lung tissue, while MDA-MB-231 cells were evaluated as breast milk is a common transmission method of AFB1 toxicity to infants. Our data revealed numerous lipid-related pathways related as altered by AFB1 across the studied cell lines. Treatment of HepG2 cells with AFB1 caused perturbations in alpha linolenic and linoleic acid metabolism, oxidation of branched chain fatty acids, carnitine synthesis, and beta oxidation of very long chain fatty acids. With the exception of alpha linolenic and linoleic acid metabolism, all of these pathways were also altered in A549 cells, and all except for beta oxidation of very long chain fatty acids were altered in MDA-MB-231 cells. Notably, catecholamine biosynthesis was also perturbed in all cell lines by AFB1 treatment. In addition to similarities between cell lines, our data also show that AFB1 effects are different for different cell lines. For example, purine metabolism as well as spermine and spermidine biosynthesis were altered in both HepG2 and MDA-MB-231 cells, but not in A549. This data supports that AFB1 can have significantly different metabolic effects depending on the tissue type, but lipid and catecholamine metabolism are most commonly affected.

There have been relatively few metabolomic studies examining the effects of AFB1. One study using HepB3 cells found that pathways associated with lipids in the cell membrane such as arachidonic acid metabolism and glycerophospholipid metabolism were altered following AFB1 treatment [49]. Upregulation of glycerophospholipid metabolism and choline metabolism were observed in goat plasma after AFB1 administration [50]. Induced mammary gland toxicity in pregnant and lactating rats also identified glycerophospholipid metabolism as an altered pathway from AFB1, as did an analysis of NCM460 intestinal cells and a lipidomics study of zebrafish brains which also found alterations in sphingolipid and glycerolipid metabolism [51–53]. These findings align with our data that identified alpha-linolenic acid and linoleic acid metabolism as altered pathways in HepG2 cells as well as phosphatidylcholine metabolism in MDA-MB-231 cells. Our data also revealed changes in various amino acid pathways following AFB1 treatment which was consistent with other metabolomic studies [49,50]. Finally, our study found that purine metabolism was altered in HepG2 cells following AFB1 treatment, a finding that was also shown previously in HepB3 cells [49].

Lipid metabolism dysregulation has been a known consequence of cancer, and treatments that target it have been a topic of research. Cancer cells need an abundance of lipids to form membranes for daughter cells, generate energy, and act as signaling molecules [54]. Metabolic studies that evaluated the effects of AFB1 commonly implicated lipid metabolism dysregulation in its mechanism to induce toxicity in the liver [55,56]. Lipid biosynthesis and desaturation has been found to be a necessary component of HCC proliferation and has suppressed cancer growth and/ or induced apoptosis when inhibited. This is evident by upregulation of fatty acid synthase, acetyl CoA carboxylase, stearoyl CoA desaturase-1, and ATP citrate lyase in HCC [57]. Carnitine metabolism was also identified in our study as a perturbed pathway. Upregulation of CPT1c has been seen in many cancers, likely as a mechanism to generate ATP and NADPH in response to glucose deprivation [58–60]. CPT1 has also been seen to have a role in cancer cell gene expression, growth, metastasis, and apoptosis in certain cell types [59]. Taken together, our data suggests that alterations in lipid metabolism may be a potential mechanism that supports AFB1-induced carcinogenesis in liver and lung cells.

Purine metabolism was an additional pathway that was significantly affected in HepG2 cells when treated with AFB1 in multiple analyses. As highlighted previously, it has been established that a mechanism in which AFB1 causes cancer is by forming AFB1-N7-guanine adducts which then form AFB1-FAPy or AP sites, causing DNA mutations. Furthermore, N6-methyladenosine (m6A) dysregulation has been observed to have a role in liver carcinogenesis, possibly indicating another mechanism in which AFB1 causes the development of HCC via altered levels of purine metabolism. Given that purines and their associated enzymes are necessary to provide the building blocks for the uncontrolled cell growth seen in cancer, it is plausible that altered purine metabolism may contribute to AFB1-induced carcinogenesis [61–63].

AFB1 and its metabolites have been established to be transmitted in the breast milk to impart toxicity on infants and sometimes cause growth impairment. The altered pathways that we observed in MDA-MB-231 cells could be reflective of the altered nutrient profile of the breast milk and contribute to the growth abnormalities associated with consuming AFB1-contaminated breast milk. Spermine and spermidine biosynthesis, carnitine synthesis, phospholipid/unsaturated fatty acid metabolism, and amino acid pathways were among the major metabolic effects of AFB1 treatment in MDA-MB-231 cells. Many of these pathways play a significant role in fatty acid beta oxidation and energy production which may affect development. Preterm infants have reduced capacity to store and synthesize carnitine and a diet containing carnitine is necessary for adequate growth [64,65]. Furthermore, breast milk is commonly the source of carnitine for infants and carnitine intake in preterm infants also has been shown to have a positive correlation with brain size, height, weight, and head circumference z-score change with very preterm infants [66,67]. If this pathway is altered in a mother’s breast cells and affects the nutritional composition of breast milk, this could play a role in growth stunting associated with AFB1 toxicity in infants.

Pathways related to plasma membrane components were also identified in many MDA-MB-231 analyses. Phosphatidylcholine biosynthesis was a very prominent pathway, but alpha linolenic acid and linoleic acid metabolism were also implicated in the analyses of MDA-MB-231 cells when treated with 5 μM of AFB1. In Malawi children with environmental enteric dysfunction, a common source of growth stunting in children of low-income countries, phospholipids such as phosphatidylcholine levels were lowered [68]. A study of undernourished rural Pakistani infants found that they had a deficiency in essential fatty acids (linoleic acid and total n-6 polyunsaturated fatty acids). Because of this, the body would counteract this effect by having high levels of oleic acid and elongase and desaturase activity used in n-3 and n-6 synthesis. This deficiency was found to be greatly associated with acute and chronic growth faltering in children with environmental enteric dysfunction [69]. AFB1 exposure is known to trigger dysfunction in the intestinal barrier as well as intestinal immune function [32,70]. Thus, changes in phosphatidylcholine and essential polyunsaturated fatty acid synthesis in the breast may participate in its deficiency in the infant, contributing to the growth stunting associated with AFB1 toxicity.

In conclusion, this study evaluated the effects of varying concentrations of AFB1 treatment (2.5 μM, 5 μM, 10 μM) on HepG2 liver cells, A549 lung cells, and MDA-MB-231 breast cells. Our analyses yielded various significantly affected metabolic pathways in each cell type–some pathways were commonly affected across all cell lines whereas other pathways were affected in a cell type-dependent manner. While the metabolic effects of AFB1 were broad, pathways related to lipid metabolism (fatty acid beta oxidation, phoshoplipid metabolism) and tyrosine metabolism (catecholamine biosynthesis) emerged as major metabolic pathways affected in all cell lines. As liver and lung cancers are known to be associated with AFB1 exposure, understanding which pathways are changed may be essential to reveal possible therapeutic targets to combat AFB1-induced carcinogenesis. Given that breast milk is a common vehicle for AFB1 exposure to infants and subsequently causes growth stunting, we analyzed the treatment of MDA-MBA-231 cells to consider how its affected pathways may affect the nutrient composition of breast milk and contribute to AFB1-related developmental toxicity. Similarly, a proper understanding of what metabolic pathways contribute to AFB1 toxicity may enable the development of targeted treatments to prevent or mitigate its adverse effects. Future research in this area includes conducting a similar investigation using animal models where the metabotypes of liver, lung, and breast tissue are evaluated. This has several advantages over *in vitro* approaches which can be influenced from experimental conditions (e.g., media conditions, types of plastics used) that can alter results. Our findings will provide a foundation for these future investigations with larger sample sizes and *in vivo* models. In addition, other key organs could also be examined such as the brain or gut epithelium to further understand tissue-specific effects of AFB1. Studies involving measuring the exometabolome of treated cells as well as circulating metabolites in breast milk or blood in AFB1-exposed individuals would also be valuable follow up studies. Additionally, future experiments should also investigate whether targeting these metabolic processes alters the cytotoxicity or genotoxicity of AFB1 in various tissues, which could potentially identify new interventions to protect against AFB1 toxicity.

## Supporting information

S1 Fig Quality control plot of metabolomics data. PCA of all study samples and quality control study pools (QCSPs) for (A) all cell lines used, (B) A549 cells only, (C) HepG2 cells only, and (D) MDA-MB-231 cells only using all metabolomics peaks.
(TIF)

S1 Table Peak statistics for all cell lines and treatment groups. (XLSX)
