# Supplementary figures and images for: An untargeted metabolomic analysis of acute AFB1 treatment in liver, breast, and lung cells
Source: PLoS One. 2025 Jan 30;20(1):e0313159. doi: 10.1371/journal.pone.0313159 (PMC11781672; doi:10.1371/journal.pone.0313159)

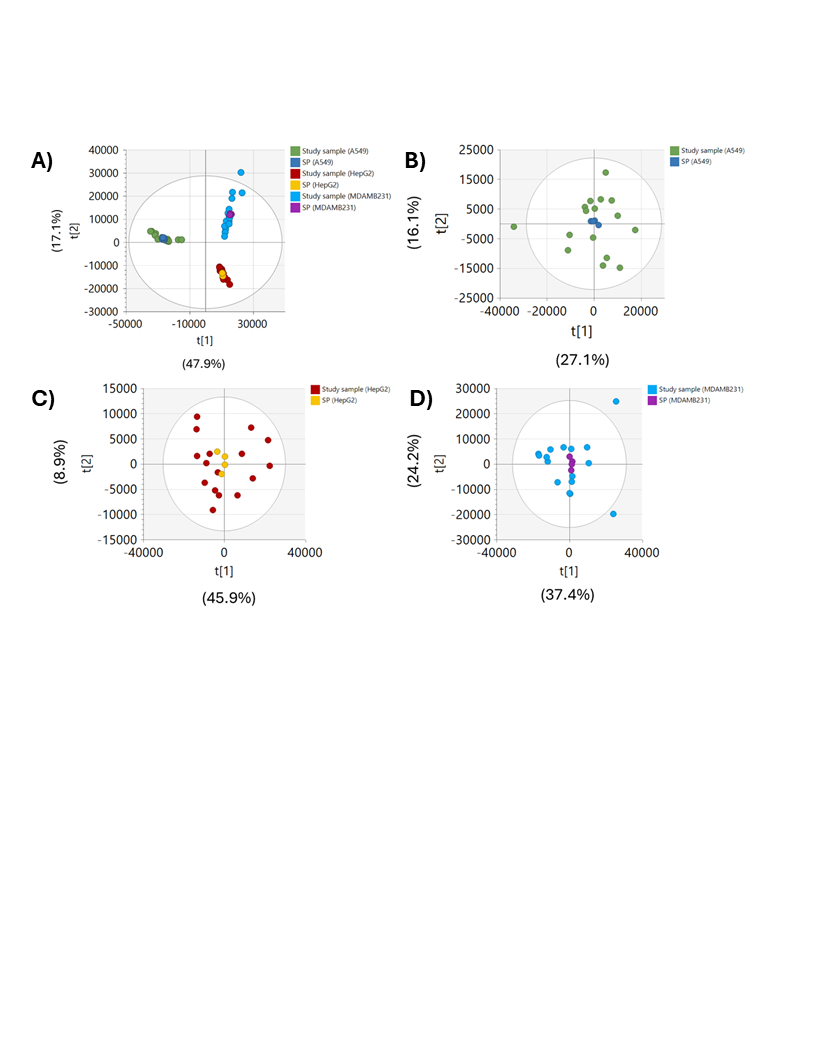

Supplement: S1 Fig — PCA of all study samples and quality control study pools (QCSPs) for (A) all cell lines used, (B) A549 cells only, (C) HepG2 cells only, and (D) MDA-MB-231 cells only using all metabolomics peaks. (TIF) [file pone.0313159.s001.tif]
